# Computed Tomography-Based Radiomics Signature for the Preoperative Differentiation of Pancreatic Adenosquamous Carcinoma From Pancreatic Ductal Adenocarcinoma

**DOI:** 10.3389/fonc.2020.01618

**Published:** 2020-08-25

**Authors:** Shuai Ren, Rui Zhao, Wenjing Cui, Wenli Qiu, Kai Guo, Yingying Cao, Shaofeng Duan, Zhongqiu Wang, Rong Chen

**Affiliations:** ^1^Department of Radiology, The Eighth Affiliated Hospital, Sun Yat-Sen University, Shenzhen, China; ^2^Department of Radiology, Affiliated Hospital of Nanjing University of Chinese Medicine, Nanjing, China; ^3^The First School of Clinical Medicine, Nanjing University of Chinese Medicine, Nanjing, China; ^4^Department of Diagnostic Radiology and Nuclear Medicine, School of Medicine, University of Maryland, Baltimore, Baltimore, MD, United States; ^5^GE Healthcare, Shanghai, China

**Keywords:** computed tomography, pancreatic neoplasms, pancreas, adenocarcinoma, adenosquamous carcinoma, radiomics

## Abstract

**Purpose:**

The purpose was to assess the predictive ability of computed tomography (CT)-based radiomics signature in differential diagnosis between pancreatic adenosquamous carcinoma (PASC) and pancreatic ductal adenocarcinoma (PDAC).

**Materials and Methods:**

Eighty-one patients (63.6 ± 8.8 years old) with PDAC and 31 patients (64.7 ± 11.1 years old) with PASC who underwent preoperative CE-CT were included. A total of 792 radiomics features were extracted from the late arterial phase (*n* = 396) and portal venous phase (*n* = 396) for each case. Significantly different features were selected using Mann–Whitney *U* test, univariate logistic regression analysis, and minimum redundancy and maximum relevance method. A radiomics signature was constructed using random forest method, the robustness and the reliability of which was validated using 10-times leave group out cross-validation (LGOCV) method.

**Results:**

Seven radiomics features from late arterial phase images and three from portal venous phase images were finally selected. The radiomics signature performed well in differential diagnosis between PASC and PDAC, with 94.5% accuracy, 98.3% sensitivity, 90.1% specificity, 91.9% positive predictive value (PPV), and 97.8% negative predictive value (NPV). Moreover, the radiomics signature was proved to be robust and reliable using the LGOCV method, with 76.4% accuracy, 91.1% sensitivity, 70.8% specificity, 56.7% PPV, and 96.2% NPV.

**Conclusion:**

CT-based radiomics signature may serve as a promising non-invasive method in differential diagnosis between PASC and PDAC.

## Introduction

Pancreatic ductal adenocarcinoma (PDAC) accounts for the majority of pancreatic malignant neoplasms, and the overall 5-year survival rate lags at 8% (1). Pancreatic adenosquamous carcinoma (PASC) is identified as a variant of PDAC, which accounts for only 1–4% of pancreatic adenocarcinoma (2), demonstrating both squamous and glandular differentiation (3, 4).

PASC patients demonstrate a slight male preponderance, with neoplasms frequently located in the head of the pancreas, which is commonly seen in PDAC patients (2). The clinical symptoms and manifestations of PASC, such as abdominal pain, body weight loss, and jaundice, are also similar to those of PDAC (3, 5), making it difficult to differentiate between the two entities. However, PASC is considered to be more aggressive than PDAC, which has a higher frequency to simultaneous metastasis to the lymph nodes and the liver (2, 6). Consequently, patients with resected PASC have a poorer prognosis than those with PDAC (median survival: 12 vs. 16 months) (2). Surgical resection with R0 margin offers the only potential chance of a cure in patients with PDAC, which is also recommended for patients with PASC (3, 7). However, patients with PASC can also benefit from radiation therapy as squamous cancer tissue is sensitive to radiation therapy. Some conventional CT or MRI imaging features, including a round and lobulated shape, cystic changes, tumor thrombus in the portal vein system, and ring-enhancement pattern, were useful in differential diagnosis between PASC and PDAC (4, 6), but the enrolled number of patients was small (4) and discriminative sensitivity was relatively low (6). Ultrasound-guided fine-needle aspiration (US-FNA), used to sample the mass, is a sensitive and safe method in the diagnosis of solid pancreatic tumors (8). However, there has been much debate about the fact that sometimes a biopsy sample could not reflect the entire extent of the phenotype of the whole tumor due to sampling errors (9). Even EUS-FNA biopsies had 12–14% false negative rates, which can cause delayed patient care (10). Therefore, accurate preoperative discrimination between PASC and PDAS using non-invasive imaging is very important for choosing the optimal treatment and for prognosis prediction.

CT has become an important non-invasive method for the diagnosis and the evaluation of different diseases, owing to its capability to reflect the biological and the physiological characteristics of different organs. CT texture analysis (CTTA) is an emerging field of investigation which is capable of assessing tissue gray-level intensity within an image and allows accurate characterization of tumors by quantification of the intra-tumoral heterogeneity (9, 11). An investigation of CTTA has been carried out in different tumors, and the original results are encouraging (12–14).

However, no studies have been conducted with CT-based radiomics signature to preoperatively differentiate PASC from PDAC. This study assessed the predictive ability of CT-based radiomics signature in differential diagnosis between PASC and PDAC.

## Materials and Methods

### Patient Selection

**FIGURE 1:**
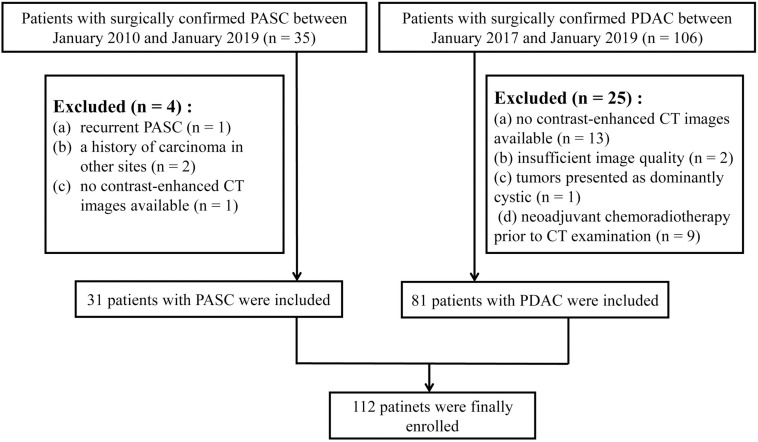
Study workflow of patient selection.

This retrospective study was approved by the Medical Ethical Committee with a waiver of patients’ approvals, which identified a total of 35 patients with pathologically confirmed PASC through surgical resection from three institutions between January 2010 and January 2019. This study also identified a total of 106 patients with pathologically confirmed PDAC through surgical resection from three institutions between January 2017 and January 2019. The inclusion criteria were as follows: (1) the patients underwent dual-phase contrast-enhanced CT (late arterial and portal venous phases) within 30 days prior to surgery, (2) the patients had a definite pathological diagnosis of PASC or PDAC, (3) the patients had optimal CT images for further radiomics analysis, and (4) the patients did not receive pre-surgical treatment such as chemoradiotherapy. Patients with PASC (*n* = 31) and PDAC (*n* = 81) were finally selected. The exclusion criteria and the acquisition of the study cohort are demonstrated in Figure 1.

### Image Acquisition

In 16 PASC patients, CE-CT was performed in our institution. In the remaining 15 PASC patients, CE-CT was performed in the other two institutions. Among the 31 PASC patients, CT was performed (a) with SOMATOM Definition (Siemens Healthcare, Germany) in 12, (b) with Optima 670 (GE Healthcare, Tokyo, Japan) in eight, (c) with Philips Brilliance 64 (Philips Healthcare, DA Best, Netherlands) in seven, and (d) with GE Lightspeed 64 VCT (GE Healthcare, United States) in four. Among the 81 PDAC patients, 63 patients underwent CE-CT in our institution (a) with Optima 670 (GE Healthcare, Tokyo, Japan) and (b) with Philips Brilliance 64 (Philips Healthcare, DA Best, Netherlands). The remaining 18 patients underwent CE-CT in the other two institutions (a) with SOMATOM Definition (Siemens Healthcare, Germany) and (b) with GE Lightspeed 64 VCT (GE Healthcare, United States). Similar protocols were adopted during the CT examinations: 120 kVp, 200–400 mAs, gantry rotation time of 0.5 s, helical pitch of 1.375, matrix of 512, and slice thickness of 1.0 mm, with a reconstruction interval of 1.0 mm. For multiphase imaging, 100–120 ml of non-ionic intravenous contrast media (Omnipaque, 350 mg I/ml, GE Healthcare) was administrated at a fixed rate of 3.0 ml/s. The scanning time delay was 40 s for the late arterial phase and 70 s for the portal venous phase.

### Technical Workflow

**FIGURE 2:**
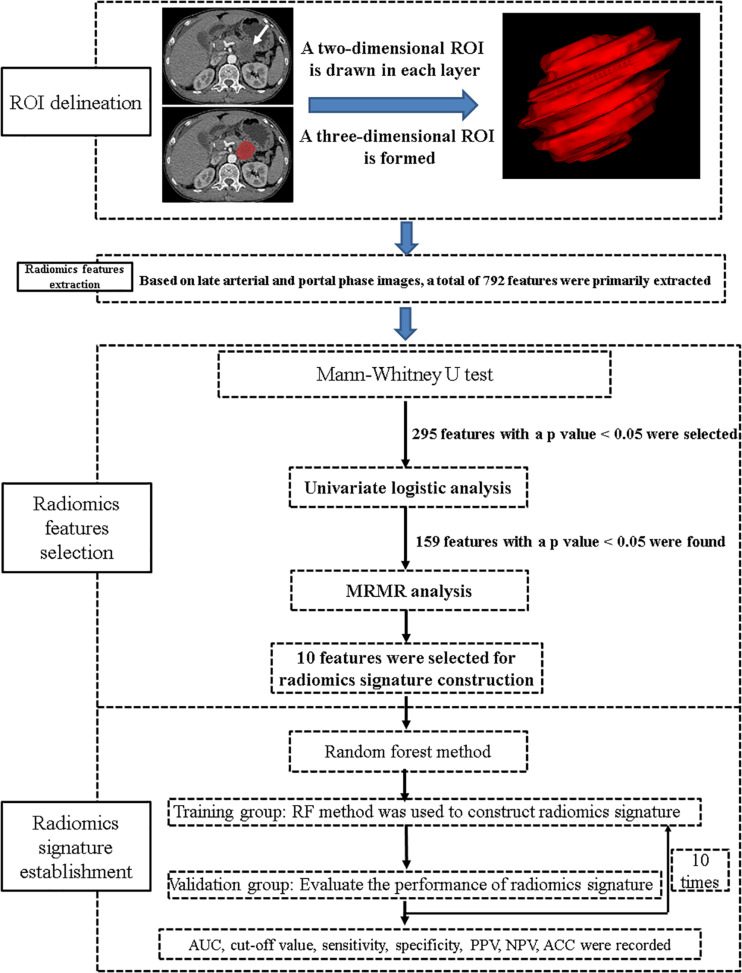
A general technical workflow of image processing and machine learning.

A general technical workflow, including tumor segmentation, radiomics feature extraction and selection, and radiomics signature construction and validation, is displayed in Figure 2.

### Tumor Segmentation

The most current NCCN guidelines (version 2.2019; April 9, 2019) recommend dual-phase pancreatic CT protocol (pancreatic phase/late arterial phase: 40–50 s; portal venous phase: 65–70 s) as necessary for the optimal evaluation of primary pancreatic tumors (7). The tumors were manually segmented from the late arterial and portal venous CT images using ITK-SNAP software (version 3.6.0)^1^. The segmentation of the tumor was based on the total volume of the tumor and done by a board-certified abdominal radiologist. The radiologist had no prior knowledge of the patients’ pathological diagnosis and had used ITK-SNAP for a prior study of 109 patients (13, 15). Moreover, during the region-of-interest delineation, the blood vessels and the lymph nodes around the tumor were avoided.

### Extraction and Selection of Radiomics Features

Two pre-processing steps were applied to CT images before feature extraction. The first one was resampling the image into 1-mm × 1-mm × 1-mm spacing to eliminate the difference of revolution and slice thickness. Second, gray level discretization was used to merge the neighboring gray levels into one level so as to eliminate the random fluctuation of the gray value, with a final 256 bins. Then, a total of 792 radiomics features were extracted from the late arterial phase (*n* = 396) and portal venous phase (*n* = 396) for each case using Analysis Kit software (version V3.0.0.R, GE Healthcare). In order to reduce the redundancy of the radiomics features, Mann–Whitney *U* test was firstly applied to explore features that are significantly different between the two groups; then, univariate logistic regression method was applied to explore the discriminative features between the two groups. Finally, minimum redundancy maximum relevance (MRMR) method (13, 15, 16), allowing the retention of the 10 most predictive radiomics features with minimum redundancy maximum relevance, was adopted.

### Construction and Validation of Radiomics Signature

Ten radiomics features, including seven features from late arterial phase images and three from portal venous phase images, were combined to construct a radiomics signature using random forest (RF) method (17), which contains a specific combination of multiple classification and regression trees comprising of independent diagnostic algorithms. The discriminative ability of the radiomics signature was recorded.

To explore the robustness and the reliability of the radiomics signature, we performed 10-times leave group out cross-validation (LGOCV) analysis (18), where the patients were randomly divided into training and testing sets with a ratio of 7:3 for 10 times. During each time, the training group was used to train a new model, and the testing set was used as an independent set with which to evaluate the model. The average performance of the 10 newly built models can be used to prove the stability and the reliability of the radiomics signature.

### Statistical Analysis

Statistical analysis was performed using SPSS version 24.0 (IBM SPSS Inc., Chicago, IL, United States) and R software, version 3.6.1^2^. A *p* value below 0.05 was considered to indicate statistical significance.

## Results

### Patient and Tumor Characteristics

Thirty-one PASC and 81 PDAC patients were analyzed in this study. The patient and tumor characteristics are listed in Table 1. No significant difference was found in terms of age, sex, tumor size, tumor location, or clinical symptoms between both groups (Table 1).

**TABLE 1:** Characteristics of patients with PASC and PDAC.

	PASC	PDAC	*P*-value
Number of patients	31	81	
Age (years)^a^	64.7 ± 11.1	63.6 ± 8.8	0.595
**Sex**			0.815
Male	18 (58.1%)	49 (60.5%)	
Female	13 (41.9%)	32 (39.5%)	
Tumor size^b^	3.75 ± 0.98 (1.9–6.7 cm)	3.51 ± 1.09 (1.5–7.0 cm)	0.293
**Tumor location**			0.554
Head and neck	21 (67.7%)	50 (61.7%)	
Body and tail	10 (32.3%)	31 (38.3%)	
Abdominal pain	14 (45.2%)	27 (33.3%)	0.245
Abdominal bloating or diarrhea	9 (29.0%)	17 (21.0%)	0.367
Body weight loss	17 (54.8%)	39 (48.1%)	0.526
Jaundice	14 (45.2%)	30 (37.0%)	0.431
Fever	3 (9.7%)	7 (8.6%)	0.863
Asymptomatic	5 (16.1%)	11 (13.6%)	0.730

*Data in parentheses are percentages. ^*a*^Data are mean age ± standard deviation. ^*b*^Data are mean diameter ± standard deviation. PASC, pancreatic adenosquamous carcinoma; PDAC, pancreatic ductal adenocarcinoma.*

### Selection of Radiomics Features

**FIGURE 3:**
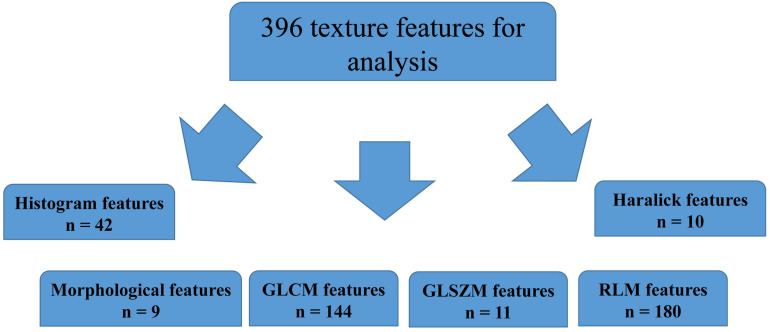
Extraction of radiomics features.

**FIGURE 4:**
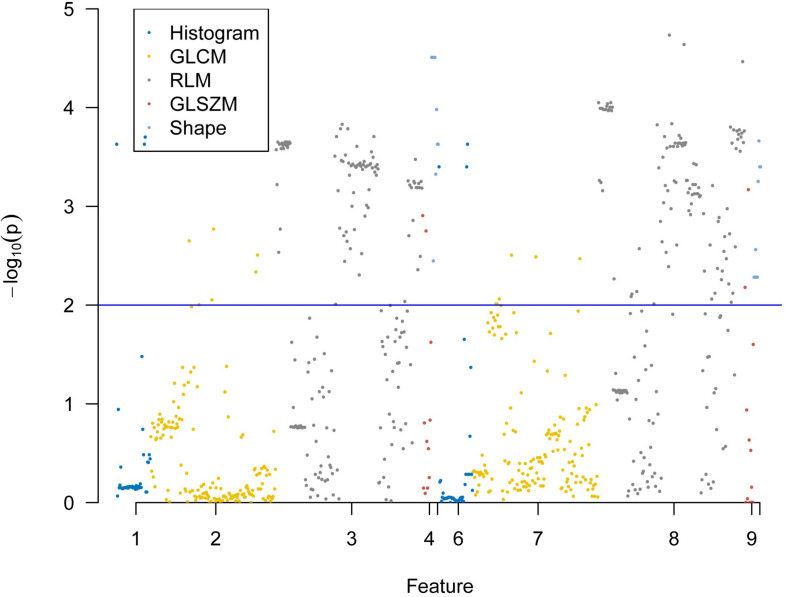
Display of *p* values between pancreatic adenosquamous carcinoma and pancreatic ductal adenocarcinoma for 792 extracted radiomics features using Manthattan.

**FIGURE 5:**
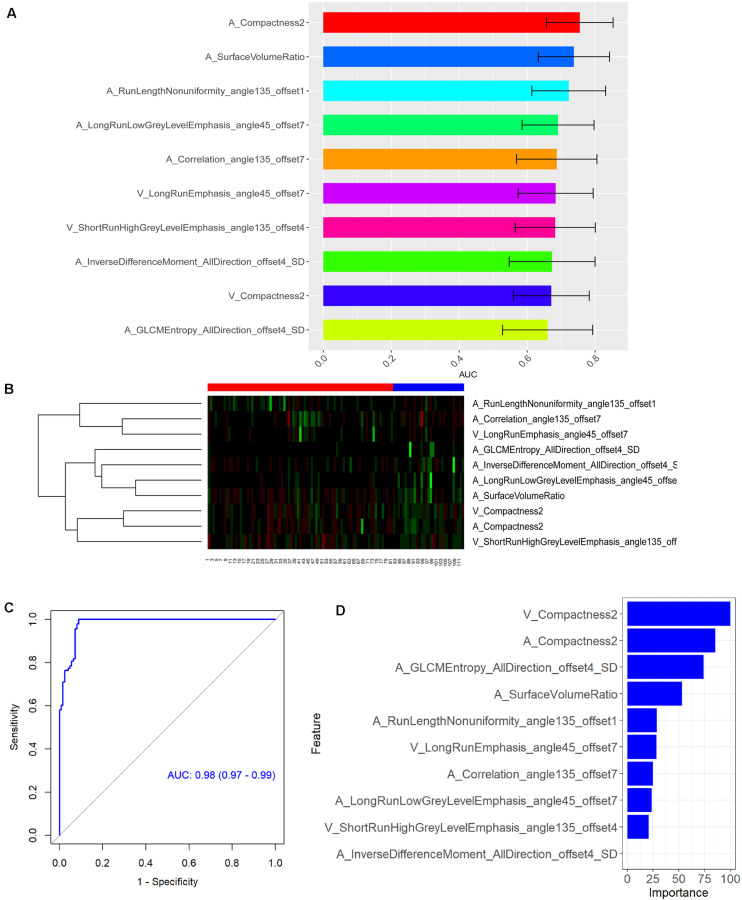
**(A)** Area under the curve barplot of the 10 selected radiomics features using minimum redundancy maximum relevance (MRMR) method. **(B)** Heat map showing the expression of the 10 selected radiomics features using MRMR method in 112 patients [numbers 1–81, pancreatic ductal adenocarcinoma (PDAC) patients; numbers 82–112, pancreatic adenosquamous carcinoma (PASC) patients]. The legend for PDAC is red color, while the legend for PASC is blue color. Regions with red intensity indicate relatively low values, while regions with green intensity represent relatively high values. **(C)** Receiver operating characteristic (ROC) curve of the CT-based radiomics signature in the differential diagnosis between PASC and PDAC. **(D)** The importance of the 10 selected radiomics features with which to construct the radiomics signature.

A total of 396 radiomics features primarily extracted from each phase CT image could be divided into six categories (Figure 3). Finally, a total of 792 radiomics features derived from dual-phase CT images were obtained and analyzed. All radiomics features were compared using Mann–Whitney *U* test between both groups, the p values of which were displayed by using Manthattan (Figure 4). As a result, a total of 295 radiomics features with a *p* value <0.05 were selected. Then, univariate logistic regression analysis was performed to explore the discriminative features between both groups, and 159 features were found. Finally, 10 radiomics features at the late arterial phase (named A_) and portal venous phase (named V_), including A_Compactness2, A_SurfaceVolumeRatio, A_RunLengthNonuniformity_angle135_offset1, A_LongRun LowGreyLevelEmphasis_angle45_offset7, A_Correlation_angle 135_offset7, V_LongRunEmphasis_angle45_offset7, V_Short RunHighGreyLevelEmphasis_angle135_offset4, A_Inverse DifferenceMoment_AllDirection_offset4_SD, V_Compactness2, and A_GLCMEntropy_AllDirection_offset4_SD, were retained using MRMR method. Two ways, including AUC barplot (Figure 5A) and heat map (Figure 5B), were used to show the remaining 10 radiomics features selected using MRMR method at the late arterial and portal venous phases. The comparisons of the 10 radiomics features between PASC and PDAC groups are summarized in Table 2. Moreover, the classification performance of each CT radiomics feature was evaluated using a receiver operating characteristic (ROC) curve (Table 3).

**TABLE 2:** Comparisons of 10 radiomics features selected using MRMR method between PASC and PDAC are expressed as median (IQR).

	PASC (*n* = 31)	PDAC (*n* = 81)	*P*-value
A_Compactness2	50,757.9 (47,172.7, 52,221.8)	47,412.1 (39,544.7, 51,009.6)	<0.001
A_SurfaceVolumeRatio	248.2 (196.8, 327.7)	159.4 (86.5, 230.5)	<0.001
A_RunLengthNonuniformity_angle135_offset1	7,151.2 (4,135.4, 14,788.0)	26,246.8 (9,207.5, 134,901.0)	<0.001
A_LongRunLowGreyLevelEmphasis_angle45_offset7	0.00034 (0.00025, 0.00054)	0.00050 (0.00031, 0.00153)	0.002
A_Correlation_angle135_offset7	0.03088 (0.01542, 0.04912)	0.00581 (−0.00261, 0.03741)	0.002
V_LongRunEmphasis_angle45_offset7	5.9 (3.9, 7.5)	8.1 (5.8, 12.4)	0.003
V_ShortRunHighGreyLevelEmphasis_angle135_offset4	52,689.5 (50,775.7, 54,904.3)	49,464.8 (41,507.5, 52,244.7)	0.003
A_InverseDifferenceMoment_AllDirection_offset4_SD	0.00011 (0.00006, 0.00018)	0.00025 (0.00011, 0.00046)	0.005
V_Compactness2	15.5 (14.0, 15.9)	14.2 (13.2, 15.1)	0.005
A_GLCMEntropy_AllDirection_offset4_SD	0.00103 (0.00041, 0.00120)	0.00354 (0.00051, 0.00729)	0.009

*MRMR, minimum redundancy maximum relevance; PASC, pancreatic adenosquamous carcinoma; PDAC, pancreatic ductal adenocarcinoma; IQR, interquartile range. Mann–Whitney *U* test was used to analyze the statistical significance between the two groups.*

**TABLE 3:** Classification performance of 10 radiomics features selected using MRMR method in the differential diagnosis between PASC and PDAC.

	AUC	Cutoff value	SEN (%)	SPE (%)	ACC (%)	PPV (%)	NPV (%)
A_Compactness2	0.755	14.3365	77.4	65.4	75.5	46.2	88.3
A_SurfaceVolumeRatio	0.738	186.403	83.9	63.0	73.8	46.4	91.1
A_RunLengthNonuniformity_angle135_offset1	0.722	16,823.4	83.9	60.5	72.2	90.7	44.8
A_LongRunLowGreyLevelEmphasis_angle45_offset7	0.691	0.0004	71.0	58.0	69.1	39.3	83.9
A_Correlation_angle135_offset7	0.687	0.0153	63.3	77.8	69.1	85.1	51.4
V_LongRunEmphasis_angle45_offset7	0.684	6.4944	67.7	67.9	68.4	84.6	44.7
V_ShortRunHighGreyLevelEmphasis_angle135_offset4	0.683	50,443.3	77.4	59.3	68.3	42.1	87.3
A_InverseDifferenceMoment_AllDirection_offset4_SD	0.673	0.0002	64.5	79.0	67.3	54.1	85.3
V_Compactness2	0.671	15.4559	51.6	84.0	67.1	55.2	81.9
A_GLCMEntropy_AllDirection_offset4_SD	0.660	0.0039	48.4	92.6	66.0	71.4	82.4

*MRMR, minimum redundancy maximum relevance; PASC, pancreatic adenosquamous carcinoma; PDAC, pancreatic ductal adenocarcinoma; AUC, area under the curve; SEN, sensitivity; SPE, specificity; ACC, accuracy; PPV, positive predictive value; NPV, negative predictive value.*

### Construction and Validation of Radiomics Signature

**FIGURE 6:**
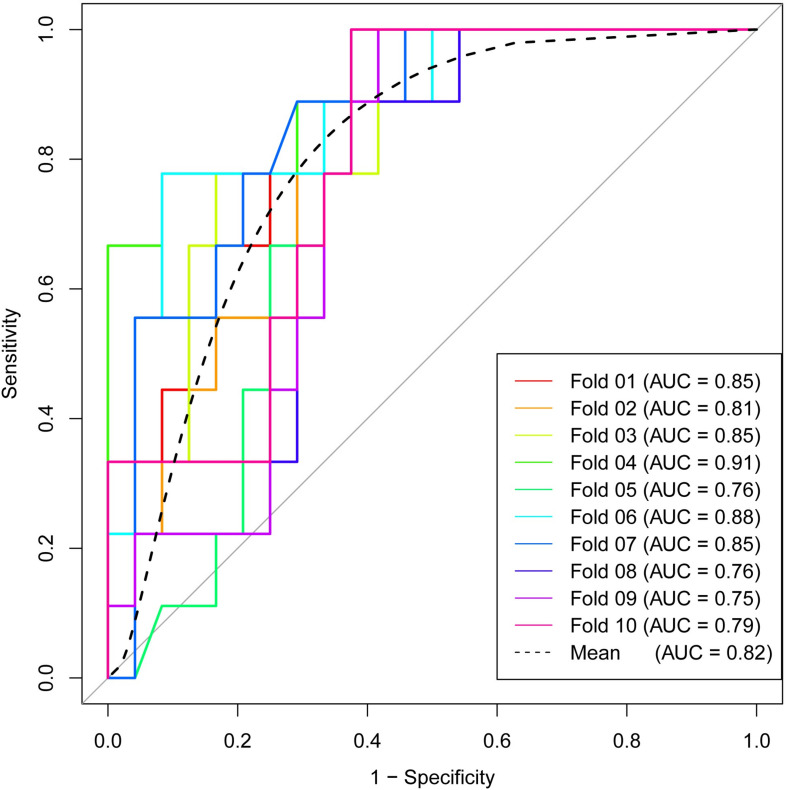
Receiver operating characteristic (ROC) curves of 10-times LGOCV analysis for differentiating pancreatic adenosquamous carcinoma from pancreatic ductal adenocarcinoma, the mean AUC of which was 0.82. The radiomics signature was proved to be robust and reliable.

Subsequently, RF method was used to construct the radiomics signature which displayed differential diagnosis ability between PASC and PDAC with 10 radiomics features. The results revealed that the radiomics signature was able to discriminate between PASC and PDAC. The classification performance of the radiomics signature had 94.5% accuracy, 98.3% sensitivity, 90.1% specificity, 91.9% PPV, and 97.8% NPV, with an AUC of 0.98 (Figure 5C). The importance of the 10 radiomics features is shown in Figure 5D. Then, we performed 10-times LGOCV analysis to explore the robustness and the reliability of the radiomics signature. The radiomics signature was proved to be robust and reliable using LGOCV method, the accuracy, sensitivity, specificity, PPV, and NPV of which were 76.4, 91.1, 70.8, 56.7, and 96.2%, with an average AUC of 0.82. Figure 6 shows the results of 10-times LGOCV analysis in differentiating PASC from PDAC using ROC curves, which proved that the radiomics signature was relatively robust and reliable.

## Discussion

PASC is a rare malignant neoplasm of the pancreas, characterized by a mixture of glandular and squamous differentiation, which is very difficult to be discriminated from PDAC (3). In a population-based analysis, it was reported that the postoperative overall survival in patients with PASC after surgical resection is significantly worse than that of patients with PDAC after surgical resection (2). An accurate preoperative diagnosis is of great importance in the patients’ prognosis prediction. There are two major obstacles in accurately differentiating between PASC and PDAC: One is that these two types of tumors share similar clinical symptoms, such as abdominal pain and jaundice (3, 5); the other is that very few features with high sensitivity and specificity on imaging studies are found in the preoperative discrimination between both groups (4, 6, 19–21).

Malignant tumors display heterogeneity with variable internal spatial organization due to differences in cellularity and angiogenesis. Tumors with highly aggressive behavior and subsequent poor prognosis have high intratumoral heterogeneity (22, 23). Evidence for such histologic characteristics could be embedded into the pixels of CT or MRI images, which could be evaluated by texture analysis, as it provides a potential method for the quantification of tumor spatial heterogeneity (9). The tumor classification of PASC and PDAC depends on the microscopic evaluation of uniformity and heterogeneity. Microscopic heterogeneity could also be reflected by tumors grossly. Interestingly, texture analysis provides a plausible method to non-invasively evaluate macroscopic heterogeneity (15, 24). Li et al. (25) have explored the potential application of CT-based texture analysis in discriminating atypical pancreatic neuroendocrine tumors (PNETs) from PDAC. D’Onofrio et al. (12) have shown the potential of 3D CT texture analysis in PNET grading. Similarly, in our previous studies, CT texture analyses have been proven to be a plausible quantitative method to differentiate between pancreatic lesions which share similar conventional imaging findings (13, 14).

Thus far, to our best knowledge, no studies have evaluated the potential value of CT-based radiomics signature to preoperatively differentiate PASC from PDAC. Toshima et al. (4) analyzed the CT and MR imaging findings in eight PASC and 33 PDAC patients. They found that PASC demonstrated a higher frequency of a round and lobulated-shaped (100 vs. 57.6%) tumor thrombus in the portal vein system (37.5 vs. 6.1%) and necrotic portions (100 vs. 39.4%) as compared with PDAC, but the number of enrolled PASC patients was relatively small. In a study by Imaoka et al. (6), they concluded that PASC showed a higher frequency of a smooth outline, ring-enhancement pattern, and cystic changes as compared with PDAC, and the most predictive feature was the ring-enhancement pattern, which showed a low sensitivity of 65.2% and a specificity of 89.6%. In our study, seven radiomics features from late arterial phase images and three from portal venous phase images were finally selected. All radiomics features showed an acceptable classification performance for differentiating PASC from PDAC, with a range from 0.660 to 0.755 of AUC. In addition, RF method was used to construct the radiomics signature, which showed 94.5% accuracy, 98.3% sensitivity, 90.1% specificity, 91.9% PPV, and 97.8% NPV, with an AUC of 0.98. Finally, 10-times LGOCV method was used to validate the robustness and the reliability of the radiomics signature. A high pooled sensitivity of 91.1%, specificity of 70.8%, and accuracy of 76.4%, with an average AUC of 0.82, indicated a stable and reliable radiomics signature.

We are aware of some limitations. First, the number of PASC patients was small for radiomics analysis due to its low incidence rate, which only accounts for 1–4% of pancreatic adenocarcinoma (2). Second, an inherent selection bias cannot be avoided due to the retrospective nature of the study. Third, different scanners were adopted. However, similar parameters were used during CT scanning, and pre-processing steps were adopted before feature extraction. Despite the use of different tube currents, a study by Mulé et al. (26) indicated that different tube currents have a little influence on the CT texture parameters. Fourth, conventional imaging features were not investigated in the study as we thought that there is appropriate evidence in the literature concerning this topic. Fifth, an external validation or a multi-center validation of these promising outcomes will be needed in the future. Finally, a core issue concerns the context in which the texture was filed as deciphered of the radiomics features, even though they were somehow validated (27).

In conclusion, our study developed and validated a CT-based radiomics signature in differential diagnosis between PASC and PDAC, which may serve as a promising non-invasive method.

## Data Availability Statement

All datasets presented in this study are included in the article/supplementary material.

## Ethics Statement

The studies involving human participants were reviewed and approved by the ethics committee of the Eighth Affiliated Hospital, Sun Yat-Sen University. Written informed consent for participation was not required for this study in accordance with the national legislation and the institutional requirements.

## Author Contributions

SR and ZW: guarantor of integrity of the entire study, funds collection, study concepts and design. RZ, WC, KG, WQ, and YC: clinical studies and literature research. RC and SD: statistical analysis. SR, ZW, and RC: manuscript editing. All authors contributed to the article and approved the submitted version.

## Conflict of Interest

SD was employed by the company GE Healthcare. The remaining authors declare that the research was conducted in the absence of any commercial or financial relationships that could be construed as a potential conflict of interest.

## Footnotes

1 http://www.itksnap.org/

2 https://www.r-project.org
